# Solubility of Amino Acids in the Eutectic Solvent Constituted by Sodium Acetate Trihydrate and Urea and in Its Mixture with Water

**DOI:** 10.3390/ijms24021550

**Published:** 2023-01-12

**Authors:** Cristina Gallego, Héctor Rodríguez, Ana Soto

**Affiliations:** Cross-Disciplinary Research Center in Environmental Technologies (CRETUS), Department of Chemical Engineering, Universidade de Santiago de Compostela, E-15782 Santiago de Compostela, Spain

**Keywords:** collagen, amino acids, solubility, eutectic, urea, sodium acetate trihydrate

## Abstract

Industrial fish and aquaculture processing leads to the generation of a huge quantity of by-products, whose accumulation and mismanagement involve serious environmental consequences as well as high economic losses. Taking advantage of these residues as a source of added-value compounds must be a priority in a circular economy. This work is a preliminary study to analyze the possibility of using the eutectic mixture of urea and sodium acetate trihydrate as a solvent for collagen extraction. To that end, the solid–liquid equilibrium of the system was determined in order to define the exact composition and temperature of the eutectic. The solubility in this solvent of the main amino acids that constitute fish collagen was studied at several temperatures and atmospheric pressure. At 308.15 K, solubilities of the major constituents of the target protein, namely L-proline, trans-4-hydroxy-L-proline, and glycine, were 0.19, 0.16, and 0.12 (mass fraction), respectively. These values increased with temperature. Dilution with water (50 wt%) allowed operation at lower temperature and led to an increase in the solubilities. The van ‘t Hoff model was satisfactorily used to correlate the experimental data and to calculate apparent properties of dissolution. All the dissolution processes studied herein are endothermic, non-spontaneous, and enthalpy-driven. Both the eutectic and its mixture with water are promising solvents for the design of an environmentally benign process for collagen extraction.

## 1. Introduction

In order to meet the growing demand for food, worldwide capture fisheries and aquaculture production have doubled in the last three decades, reaching a value of 180 Mt/year. This expansion in the size of fish-processing industrial capacity is leading concomitantly to an increase in the amount of the low-value by-products generated, which may represent up to 70% of the total mass processed, depending on factors such as the species, the final product, or the type of processing [1]. Such by-products are mainly composed of heads, skin, viscera, bones, or scales, and they have been traditionally diverted to the production of fishmeal, animal feed, or fertilizer, or they have even been simply discarded as waste. In addition to the direct impact on the environment and thus on human health, this strategy also involves tacitly high economic losses, since the aforementioned by-products have the potential to be a valuable source of bioactive compounds [2]. Therefore, in the context of sustainable management of natural resources and rapid growth in public awareness about sustainability, the utilization of fish by-products as a raw material to recover valuable components is emerging as a topic of great interest [3].

One of the most interesting compounds contained in fish by-products is collagen, which is the main structural protein in extracellular connective tissues of the fish body, constituting 15–30% of the total protein content. Although collagen has been classified into at least 28 types based on molecular weight, amino acid composition, or functionality, the most common is the so-called collagen type I, mainly composed of three amino acids: glycine, proline, and hydroxyproline. There is a high demand for collagen type I in biomedicine, pharmaceutical, cosmetic, food, and biomaterial industries due to its biodegradability, biocompatibility, and antigenicity [4,5,6]. Hydrogen bonds in collagen can be broken upon denaturation through thermal or chemical treatments, significantly affecting its properties and resulting in another widely known product of commercial interest: gelatin.

In an attempt to make extraction processes greener and more effective, eutectics are currently gaining much interest as alternative solvents for recovering bioactive molecules from different food matrices, including fish by-products [7,8]. A eutectic reaction is defined as an isothermal reversible reaction between two (or more) solid phases during the heating of a system, as a result of which a single liquid phase is produced [9]. The eutectic composition has a melting temperature lower than that of any of the parent compounds, enabling its use as a liquid solvent at temperatures at which the individual consideration of the higher-melting parent compounds as solvents would not be possible due to their solid character. Since Abbott’s pioneering work [10,11], unfortunately, the meaning of a eutectic solvent has been perverted by the prolific utilization of the term “deep eutectic solvent” to refer to many liquid mixtures without evidence of eutectic behavior supported by the corresponding solid–liquid equilibria. Without intending to neglect the practical interest that composed solvents can have for many varied applications, the work carried out by Coutinho and coworkers aiming to correct some widespread misconceptions in the (ab)use of the label “deep eutectic solvent” must be highlighted [12,13,14]. In that vein, the adjective “deep” should be restricted to eutectic mixtures for which the melting temperature of the eutectic composition would be lower (how much lower is considered low enough is yet to be shaped by the scientific community through pertinent debate) than one corresponding to the ideal behavior of the liquid mixture. In any case, before referring to a solvent as (deep) eutectic, the solid–liquid equilibrium must be determined in order to verify the eutectic behavior and the corresponding melting temperature and composition of the eutectic point.

Besides enabling high-melting compounds to be used in liquid form as part of the solvent, for example in extraction processes, a further advantage of eutectic solvents is their tunability, since it is possible to select the corresponding parent compounds depending on the desired application. Focusing on the extraction of bioactive compounds, the use of eutectic solvents resulting from the combination of GRAS (“generally regarded as safe”) compounds sounds attractive.

A number of eutectics with a melting temperature below or just slightly above room temperature have been investigated in the literature for the recovery of components of interest from bio-residues. Most of these are based on the combination of (2-hydroxyethyl)trimethylammonium chloride ([choline]Cl) with different organic acids, as in the work by Bradić et al. [15], who paired [choline]Cl with lactic, malonic, and citric acid to obtain chitin from shrimp shells. In a similar vein, although in this case for extracting collagen peptides, Bai et al. [16] combined [choline]Cl with lactic, oxalic, and acetic acids, as well as with other organic compounds such as urea, ethylene glycol, or glycerol, to generate eutectic solvents. Bisht et al. [17] tested aqueous solutions of several eutectic solvents to extract collagen from the skin of Atlantic cod, with the system containing urea and lactic acid leading to the best performance, improving on the traditional method with acetic acid. Regarding the eutectic mixture composed of sodium acetate and urea, Nuutinen et al. [18] reported its effectiveness as a solvent for extracting keratin from poultry feathers when diluted with water. The same mixture was successfully used by Rico et al. [19] to valorize melon by-products.

**Figure 1:**
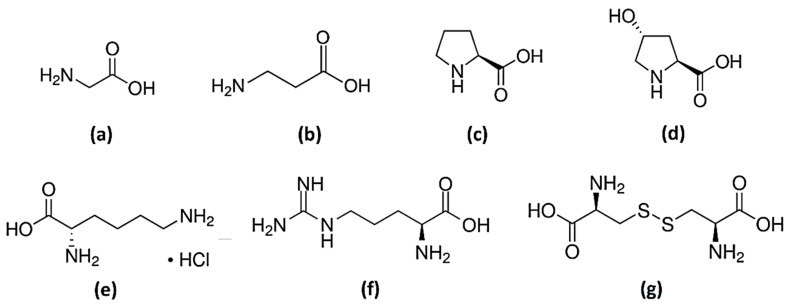
Chemical structures of amino acids: (**a**) glycine (Gly); (**b**) β-alanine (Ala); (**c**) L-proline (Pro); (**d**) trans-4-hydroxy-L-proline (OH-Pro); (**e**) L-lysine monohydrochloride (Lys·HCl); (**f**) L-arginine (Arg); and (**g**) L-cystine (Cys).

This work aims to be a preliminary study on the use of a eutectic system based on GRAS components for the extraction of collagen from fish residues. Sodium acetate trihydrate was selected as a first component due to the basic character of the acetate anion, which is expected to be able to disrupt the hydrogen-bonding network of the constitutive biopolymers of fish residues. In fact, the acetate anion has proven its capacity to recover bioactive compounds from fish waste as part of ionic liquids [20,21,22]. Urea was selected as the second component due to its frequent use in the biopharmaceutical industry to solubilize proteins. After ascertaining the eutectic behavior and characteristics, the solubility of representative amino acids of fish collagen and gelatin (see Figure 1) will be determined in this solvent. The possibility of using a ternary mixture comprising the eutectic compounds and water will also be analyzed through the corresponding solubility studies. As far as we know, and despite the huge interest of these combinations, this is the first study about solubilities of amino acids in eutectics.

## 2. Results and Discussion

### 2.1. Determination of the Eutectic

Samples of urea and of sodium acetate trihydrate were independently analyzed by differential scanning calorimetry (DSC) to determine their melting temperatures and enthalpies of fusion. Experimental results are shown in Table 1 along with values reported in the literature. A reasonable agreement can be observed between both sets, with the minor discrepancies being attributable to different experimental conditions during the measurements, to the presence of different types and levels of impurities, or to the moisture of the samples.

**Figure 2:**
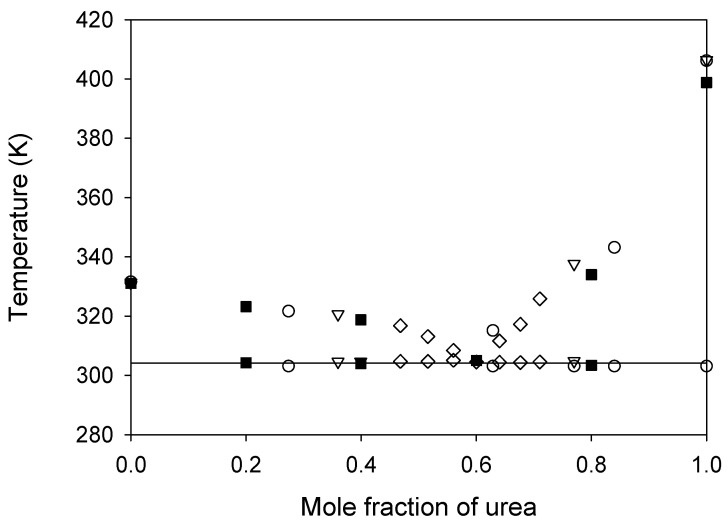
Temperature–composition diagram for the solid–liquid equilibrium of the urea + sodium acetate trihydrate system. Experimental results (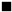) are compared to literature data: 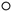, ref. [24]; 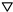, ref. [28]; 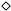, ref. [29]. The horizontal solid line represents the eutectic temperature found.

Both urea and sodium acetate trihydrate melt at temperatures considerably above ambient conditions, thus limiting their practical use as solvents for a number of applications. The existence of eutectic behavior in this system would overcome this issue, expanding the liquid range of the mixture while fully keeping the properties of its constituents. The solid–liquid equilibrium of this system has been previously studied [24,28,29]. However, some discrepancies were found regarding the eutectic composition. Thus, the urea + sodium acetate trihydrate system was investigated via DSC to dispel doubts on its solid–liquid equilibrium and ascertain the characteristics of the eutectic point. The temperature–composition plot is shown in Figure 2, which includes the previously available literature data for comparison purposes. As expected, a eutectic state of solid–solid–liquid equilibrium was found. The urea mole fraction at the eutectic is 0.60 and the eutectic temperature is 304 K. This represents a relevant depression of the melting temperature with respect to those of the individual constituents, thus allowing the mixture to be used as a solvent at extraction temperatures very close to ambient conditions. Comparing our results for the eutectic point with those found in the literature (see Figure 2 and Table 2), the only discrepancy is found with data from Li et al. [24], who obtained a eutectic composition with a much higher urea concentration.

### 2.2. Solubility Studies

**Figure 3:**
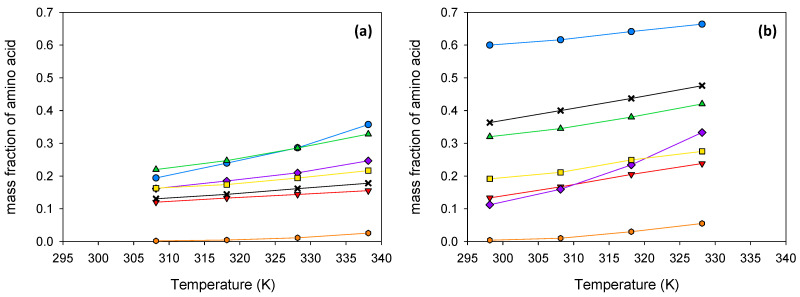
Solubilities of amino acids in the eutectic solvent (**a**) and in its 50:50 (wt:wt) mixture with water (**b**). Results are expressed as mass fraction. Legend: 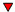 glycine; 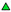 β-alanine; 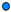 L-proline; 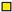 trans-4-hydroxy-L-proline; 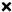 L-lysine·HCl; 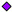 L-arginine; 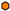 L-cystine.

The solubility of several amino acids (see Figure 1) was determined in the urea + sodium acetate trihydrate mixture of eutectic composition, as well as in its 50:50 (wt:wt) mixture with water. The addition of water allows the reduction in the viscosity of the solvent and the possibility of working at lower temperatures without risk of solidification of the eutectic-forming compounds. Unfortunately, the solid–liquid equilibrium of the ternary system was only partially studied in the literature [28]. As the study was considered to be beyond the scope of this work, a water content of 50 wt% was estimated as the limit of interest in practical applications and selected instead of the ternary eutectic. Experiments were carried out at four different temperatures, from 308.15 K to 338.15 K when using the eutectic, and from 298.15 K to 328.15 K in the case of the aqueous mixture. All the results, expressed as the mass fraction of amino acid, are shown in Table 3 and represented in Figure 3.

The urea + sodium acetate trihydrate mixture is characterized by the intensive H-bonding interactions between its components [30]. Thus, the solubilities of amino acids in this mixture can be favored, among other interactions, by the H-bonds between the solute and the solvent components [31]. At temperatures close to ambient conditions, the solubilities of the studied amino acids in the eutectic follow the trend Ala > Pro > Arg ~ OH-Pro > Lys·HCl > Gly > Cys. Cystine is a dimer of two cysteine molecules connected via a disulfide bridge. The high hydrophobicity index, estimated in terms of the solvent-accessible surface area of the disulfide-bonding amino acid [32,33], may explain a much lower solubility than the other amino acids. When water is introduced into the system, the hydrogen bonds among the eutectic constituents are gradually broken, accompanied by the formation of new bonds between water and the eutectic constituents. In the case of a significant quantity of water, the hydrogen bond network of the eutectic is destroyed, and the increase in amino-acid solubilities is due to the presence of water [30]. For this reason, it is not strange that the solubilities of the studied biomolecules in the ternary mixture (urea + sodium acetate trihydrate + water) follow the same trend as in water [34], decreasing according to Pro > Lys·HCl > Ala > OH-Pro > Gly > Arg > Cys. All the solubilities, in the eutectic and its mixture with water, increase with temperature, with this effect being particularly noticeable in the case of the solubility of L-arginine in the aqueous mixture.

Some similarities were found for the solubilities in the eutectic and in the eutectic–water mixture. In both cases, the solubility of glycine (with no hydrophobic side chain) is lower than that of β-alanine; the solubility of trans-4-hydroxy-L-proline is lower than that of L-proline, so the presence of an OH group in the proline ring results in a decrease in solubility; and the existence of a disulfide group in the amino acid (L-cystine) leads to the lowest solubilities among all the amino acids studied. However, some differences must also be highlighted. The polar portions of the L-proline molecule are sufficiently hydrated to ensure the highest solubility in the aqueous solution. However, in the case of the eutectic and at the lowest temperatures, β-alanine is more soluble than L-proline. The second most soluble amino acid in the aqueous mixture is the monohydrochloride form of L-lysine. Nonetheless, its solubility in the eutectic is rather limited.

Focusing on collagen extraction from fish residues as the application, the use of the eutectic mixture is required to make the dissolution of fish biopolymers possible through the disruption of hydrogen bonding, thus facilitating the extraction of components of interest. However, the ternary mixture with water would improve the mass transfer process due to the significant reduction in the viscosity of the solvent and, according to the solubilities here obtained, could favor an increase in the solubility of the protein in the solvent. New solubility studies involving mixtures of amino acids in the compositions found in collagens, and also the protein itself, would be the next stage of fundamental research prior to the optimization of the application.

The mathematical expression of the van ‘t Hoff equation was used for data correlation:

(1) $$\ln x=A+\frac{B}{T\left(K\right)}$$

where *x* is the solubility of the amino acid in the eutectic or its 50:50 (wt/wt) mixture with water, expressed as mole fraction; A and B are the correlation parameters; and *T* is the absolute temperature.

**Figure 4:**
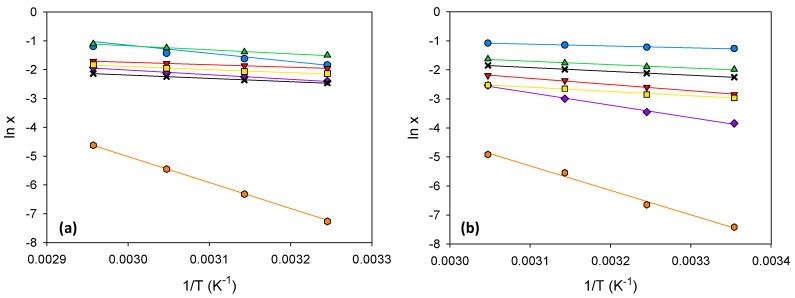
Experimental solubility data correlated by the van ‘t Hoff equation for different amino acids in the eutectic solvent (**a**) and in its 50:50 (wt:wt) mixture with water (**b**). Legend: 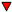 glycine; 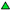 β-alanine; 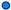 L-proline; 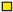 trans-4-hydroxy-L-proline; 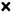 L-lysine·HCl; 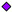 L-arginine; 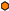 L-cystine.

Table S1 in the Supplementary Materials presents the numerical values of the solubilities expressed in mole fractions. Table 4 shows the correlation parameters and the absolute relative deviation percent (%ARD) of the linear fits of the natural logarithm of *x* versus the inverse of *T*. Figure 4 shows these linear fits together with the corresponding experimental solubility data points. It can be seen that, in all cases, this simple and classical model correlates satisfactorily to the experimental data.

#### Apparent Properties of Dissolution

The apparent standard enthalpy change for the dissolution of the amino acids in the eutectic solvent or in its 50:50 (wt/wt) mixture with water could be determined from the slope of the modified van ‘t Hoff equation [35,36,37]:

(2) $$\frac{\partial \ln x}{\partial {\left(\frac{1}{T}-\frac{1}{T_{hm}}\right)}_{p}}=-\frac{\Delta H_{d}^{0}}{R}$$

where *R* is the ideal gas constant, $\Delta H_{d}^{0}$ is the apparent standard enthalpy change of dissolution, subscript *p* denotes constant pressure, and *T_hm_* is the harmonic mean temperature calculated as:

(3) Thm=n∑i=1n1Ti

with *n* corresponding to the total number of experimental temperatures investigated. In this study, *T_hm_* = 322.76 K for the studies with the eutectic, and *T_hm_* = 312.75 K for the studies with the aqueous formulation of the eutectic.

The apparent standard Gibbs energy ($\Delta G_{d}^{o}$) and entropy changes ($\Delta S_{d}^{o}$) of dissolution can be calculated with the following equations:

(4) $$\Delta G_{d}^{o}=-RT_{hm}Intercept$$

(5) $$\Delta S_{d}^{o}=\frac{\Delta H_{d}^{o}-\Delta G_{d}^{o}}{T_{hm}}$$

where *Intercept* refers to the intercept derived from Equation (2). Table 5 shows the apparent properties of dissolution and the contributions of enthalpy and entropy to the Gibbs energy in the dissolution process calculated as:

(6) $$\xi_{H}^{d}=\frac{\left|\Delta H_{d}^{o}\right|}{\left|\Delta H_{d}^{o}\right|+T_{hm}\left|\Delta S_{d}^{o}\right|\;}$$

(7) $$\xi_{TS}^{d}=\frac{T_{hm}\left|\Delta S_{d}^{o}\right|}{\left|\Delta H_{d}^{o}\right|+T_{hm}\left|\Delta S_{d}^{o}\right|\;}$$

**Figure 5:**
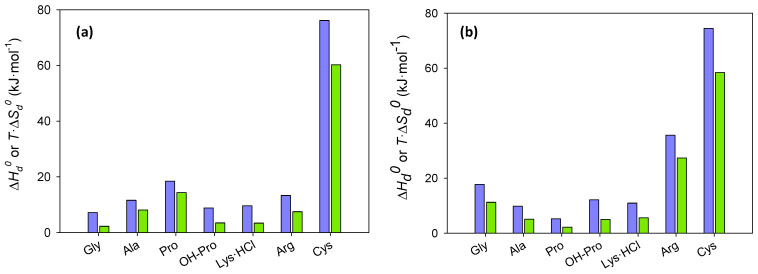
Enthalpic ($\Delta H_{d}^{o}$, blue/left bars) and entropic (*T_hm_*·$\Delta S_{d}^{o}$, green/right bars) contributions to the dissolution process in the eutectic solvent (**a**) and in its 50:50 (wt:wt) mixture with water (**b**).

As clearly shown in Table 5 and Figure 5, $\Delta H_{d}^{o}$ and $\Delta G_{d}^{o}$ of the dissolution of the amino acids in both the eutectic and its mixture with water are positive, indicating that the process is endothermic and non-spontaneous, which is in agreement with the increase in solubility with temperature. Moreover, when comparing $\Delta G_{d}^{o}$ with the solubilities shown in Tabls S1 (in mole fractions), an inverse correlation is observed, as expected. Figure 5 and the contribution parameters presented in Table 5 indicate that the enthalpy is the main contributor to $\Delta G_{d}^{o}$. However, in some cases, such as the dissolution of L-proline, L-cystine, and β-alanine in the eutectic or of L-arginine and L-cystine in the ternary mixture, contributions are rather balanced.

## 3. Materials and Methods

### 3.1. Materials

Sodium acetate trihydrate (>99 wt%) was purchased from Scharlau (Sentmenat, Spain). Urea (>99.5 wt%), glycine (>99 wt%), β-alanine (>99 wt%), trans-4-hydroxy-L-proline (>99 wt%), L-lysine monohydrochloride (>99.5 wt%), L-arginine (>98 wt%), and L-cystine (>98 wt%) were supplied by Sigma-Aldrich (Madrid, Spain). L-Proline, with purity >99 wt%, was supplied by Panreac (Castellar del Vallès, Spain). Table S2 in Supplementary Materials shows sources and purities of the amino acids used in this work. All reactants were used as received, without further purification. Bidistilled water was used throughout the experimental work in preparing the aqueous solutions.

### 3.2. Methods

#### 3.2.1. Determination of the Eutectic

Samples with a molar composition step of ca. 0.20 were prepared in glass vials, covering the entire composition range of the urea + sodium acetate trihydrate system, and evenly heated to 343 K in an oil bath until total liquefaction and homogenization were achieved. The weighing was carried out on a Mettler Toledo XPE205 analytical balance with a precision of 10^−4^ g. Approximately 5–15 mg of each sample were placed in a hermetically sealed aluminum pan and analyzed by DSC in a Q2000 differential scanning calorimeter (TA Instruments, Cerdanyola del Vallès, Spain) coupled with an RCS 90 cooling system. Each sample was loaded into the measuring chamber with an autosampler, along with an analogous empty capsule used as a reference. A 50 mL/min nitrogen flow was used as sample purge gas. The thermal program consisted in a rapid cooling to 283.15 K followed by three cycles, each of them comprising a heating ramp up to 403.15 K and a cooling ramp down to 203.15 K, at heating/cooling rates of 5 K/min, with 10 min isotherms in between these ramps. After the first heating ramp, all samples became supercooled, and no further identification of endothermic peaks connected with sample melting could be identified in the subsequent heating ramps. For this reason, the thermogram of the first heating ramp was used to determine the melting temperature (with an estimated uncertainty of 1 K) at the onset of the observed endothermic peaks. The analysis of the thermograms was carried out with the software Universal Analysis 2000, version 4.5.0.5, by TA Instruments.

#### 3.2.2. Solubility Studies

The solubility of different amino acids was measured in the eutectic solvent, as well as in its 50:50 (wt/wt) mixture with water. Jacketed glass cells were used, and the temperature was controlled by means of the circulation of water from an Ultratherm-200 P Selecta bath through the cell jackets. For each desired temperature, the solvent was placed together with an excess of the amino acid in the cell and the mixture was magnetically stirred for 24 h and then left to stand for at least 1 h (preliminary tests were carried out to ensure that these times were sufficient to achieve equilibrium conditions). After this, the supernatant was partially taken with a syringe and filtered with nylon filters (pore size 0.45 µm) to ensure removal of any non-dissolved amino acid particles. Both syringe and filters were pre-heated to ensure the absence of amino acid precipitation. Measurement of a physical property was used as the analysis method.

In the case of solubilities in the ternary mixture (eutectic:water, 50:50 wt:wt), the measurement of density was carried out for composition determination. To that end, a vibrating U-tube DMA 5000 density meter (Anton Paar, Madrid, Spain) with automatic viscosity correction and internal temperature control was used. First, several samples with amino acid concentration ranging from zero to a composition close to its solubility were prepared by weight. Then calibration curves were obtained by measuring the density of those samples. Data and correlation curves are presented in the Supplementary Materials (Tables S3–S9). To ensure the validity of this analysis method, new samples were prepared by weight and the composition calculated with the calibration curves, and results were found in good agreement within the uncertainty of the method. Second, to determine solubilities, the equilibrium samples were appropriately diluted (a mass of solvent twice that of the sample was added) for compositional analysis using the density calibration curve. The composition obtained was obviously corrected according to the mass of solvent added.

In the case of the eutectic mixture, and due to the small variation of density with amino acid content, the same analysis method led to high deviations. Therefore, refractive index was selected as the reference property for the calibration curves. An Abbemat 500 Refractometer (Anton Paar, Madrid, Spain) with embedded temperature control was used. The refractive indices of the amino acid solutions in the eutectic solvent and the calibration curves are also presented in the Supplementary Materials (Tables S3–S9). As in the previous case, the dilution of the equilibrium samples was required, and a dilution factor 1:2 with solvent was used.

## 4. Conclusions

In this work, a study regarding the solubility of different amino acids (namely: glycine, β-alanine, L-proline, trans-4-hydroxy-L-proline, L-lysine·HCl, L-arginine, and L-cystine), being representative constituents of fish collagen, was carried out. The solubilities of these biomolecules were determined in the urea + sodium acetate trihydrate eutectic and its 50:50 (wt/wt) mixtures with water at several temperatures. A discrepancy found in previous studies regarding the composition of the eutectic was solved, identifying a urea mole fraction of 0.60 as the composition that led to the lowest melting temperature of the system (304 K). This value, close to ambient conditions, is promising for the use of the eutectic as solvent in different extraction processes without the need for significant energy input to keep the solvent a liquid.

The solubilities of the most abundant amino acids in fish collagen and gelatin (glycine, proline, and hydroxy-proline) in the aforementioned eutectic were found to be rather high and increased with an increase in temperature. The capacity of the eutectic to establish hydrogen bond interactions along with its capacity to dissolve the target amino acids makes it, in principle, an interesting solvent to disrupt the hydrogen-bonding network in the biopolymers matrix of fish residues, thus potentially facilitating the extraction of the component of interest. When the eutectic was mixed with water, the solubilities of the amino acids in the solvent increased. A favored mass transfer process (viscosity reduction) and higher solubilities also encourage future studies using the ternary mixture as a possible extraction solvent.

All the dissolution processes studied herein were endothermic, non-spontaneous, and enthalpy-driven, although with quite balanced enthalpy–entropy contributions in some cases. Future studies, supported by molecular simulation, are encouraged in order to have a better understanding of how the amino acids are solvated in the eutectic medium.

## Figures and Tables

**Table 1:** Melting temperature (*T_m_*) and enthalpy of fusion (Δ*H_fus_*) of the eutectic compounds. “Exp.” refers to experimentally determined values, and “Lit.” refers to literature values.

Compound	*T_m_* (K)		Δ*H_fus_* (kJ·mol^−1^)	
	Exp.	Lit.	Exp.	Lit.
Urea	402	406 [23,24]	12.7	14.1 [25], 15.1 [24]
Sodium acetate trihydrate	331	331 [26,27], 332 [24]	37.3	37.0 [27], 36.2 [24]

**Table 2:** Urea + sodium acetate trihydrate eutectic composition and melting temperature.

Eutectic Composition (Urea Mole Fraction)	*T_m_* (K)	Reference
0.77	303	[24]
0.60	305	[28]
0.60	305	[29]
0.60	304	This work

**Table 3:** Solubilities (mass fraction) of different amino acids in the eutectic solvent and in its mixtures with water, 50:50 (wt:wt), at different temperatures and 0.1 MPa *.

			**Eutectic**				
*T* (K)	Glycine	β-Alanine	L-Proline	trans-4-Hydroxy-L-proline	L-Lysine·HCl	L-Arginine	L-Cystine
308.15	0.1201	0.2199	0.1941	0.1629	0.1307	0.1618	0.0019
318.15	0.1329	0.2471	0.2393	0.1739	0.1440	0.1850	0.0047
328.15	0.1439	0.2858	0.2864	0.1939	0.1615	0.2100	0.0113
338.15	0.1553	0.3282	0.3573	0.2166	0.1781	0.2467	0.0257
			**Eutectic:Water 50:50 (wt:wt)**				
*T* (K)	Glycine	β-Alanine	L-Proline	trans-4-Hydroxy-L-proline	L-Lysine·HCl	L-Arginine	L-Cystine
298.15	0.1334	0.3202	0.5999	0.1916	0.3633	0.1124	0.0040
308.15	0.1671	0.3448	0.6161	0.2113	0.4000	0.1596	0.0102
318.15	0.2049	0.3801	0.6412	0.2492	0.4371	0.2341	0.0300
328.15	0.2381	0.4204	0.6639	0.2753	0.4760	0.3331	0.0552

* Uncertainties: u(T) = 0.1 K. u(P) = 5 kPa. Eutectic: u_r_(w) = 0.004 Gly; u_r_(w) = 0.007 Ala; u_r_(w) = 0.005 Pro; u_r_(w) = 0.006 OH-Pro; u_r_(w) = 0.007 Lys; u_r_(w) = 0.006 Arg; u_r_(w) = 0.009 Cys. Eutectic:water: u_r_(w) = 0.003 Gly; u_r_(w) = 0.003 Ala; u_r_(w) = 0.006 Pro; u_r_(w) = 0.004 OH-Pro; u_r_(w) = 0.005 Lys; u_r_(w) = 0.003 Arg; u_r_(w) = 0.008 Cys.

**Table 4:** Regression parameters of the van ‘t Hoff model obtained from the correlation of the natural logarithm of the experimental solubility data with the inverse of the absolute temperature.

	Eutectic			Eutectic:Water 50:50 (wt:wt)		
Amino Acid	A	B (K)	% ARD	A	B (K)	% ARD
Glycine	−863.5	0.848	0.34	−2133	4.319	1.12
β-Alanine	−1395	3.014	0.94	−1181	1.957	1.45
L-Proline	−2219	5.355	1.12	−629.7	0.835	0.66
trans-4-Hydroxy-L-proline	−1064	1.300	1.55	−1492	2.023	1.63
L-Lysine·HCl	−1154	1.275	0.47	−1317	2.162	0.35
L-Arginine	−1604	2.798	1.35	−4286	10.50	3.07
L-Cystine	−9160	22.46	0.31	−8959	22.47	7.32

**Table 5:** Apparent properties of dissolution.

Amino Acid	$\Delta H_{d}^{o}\;(\mathrm{kJ}\cdot {\mathrm{mol}}^{-1})$	$T_{hm}\cdot \Delta S_{d}^{o}\;(\mathrm{kJ}\cdot {\mathrm{mol}}^{-1})$	$\Delta G_{d}^{o}\;(\mathrm{kJ}\cdot {\mathrm{mol}}^{-1})$	$\xi_{H}^{d}$	$\xi_{TS}^{d}$
		**Eutectic**			
Glycine	7.18	2.28	4.90	0.76	0.24
β-Alanine	11.60	8.09	3.51	0.59	0.41
L-Proline	18.45	14.37	4.08	0.56	0.44
trans-4-Hydroxy-L-proline	8.85	3.49	5.36	0.72	0.28
L-Lysine·HCl	9.60	3.42	6.18	0.74	0.26
L-Arginine	13.34	7.51	5.83	0.64	0.36
L-Cystine	76.16	60.28	15.87	0.56	0.44
	**Eutectic:Water 50:50 (wt:wt)**				
Glycine	17.74	11.23	6.51	0.61	0.39
β-Alanine	9.82	5.09	4.73	0.66	0.34
L-Proline	5.24	2.17	3.06	0.71	0.29
trans-4-Hydroxy-L-proline	12.40	5.26	7.15	0.71	0.29
L-Lysine·HCl	10.95	5.62	5.33	0.66	0.34
L-Arginine	35.63	27.30	8.34	0.57	0.43
L-Cystine	74.49	58.42	16.07	0.56	0.44

## Data Availability

Not applicable.

## Supplementary Materials

The supporting information can be downloaded at: https://www.mdpi.com/article/10.3390/ijms24021550/s1.
