# Supplementary material for: Solubility of Amino Acids in the Eutectic Solvent Constituted by Sodium Acetate Trihydrate and Urea and in Its Mixture with Water
Source: Int J Mol Sci. 2023 Jan 12;24(2):1550. doi: 10.3390/ijms24021550 (PMC9867164; doi:10.3390/ijms24021550)
Supplement: Supplementary file 1 [file ijms-24-01550-s001.zip › ijms-2116090-supplementary.pdf]

SOLUBILITY OF AMINO ACIDS IN THE EUTECTIC SOLVENT CONSTITUTED BY SODIUM ACETATE TRIHYDRATE AND UREA AND ITS AQUEOUS FORMULATION

Cristina Gallego, Héctor Rodríguez, Ana Soto*

CRETUS, Department of Chemical Engineering, Universidade de Santiago de Compostela, E-15782, Santiago de Compostela, Spain.

SUPPLEMENTARY MATERIALS

* Corresponding author. E-mail: ana.soto@usc.es

Table S1. Solubilities (mole fraction) of different amino acids in the eutectic solvent and in its mixture with water, 50:50 (wt:wt), at different temperatures and 0.1 MPa.

Eutectic							
T (K)	Glycine	β-Alanine	L-Proline	trans-4-Hydroxy-L-proline	L-Lysine·HCl	L-Arginine	L-Cystine
308.15	0.1411	0.2223	0.1590	0.1182	0.0850	0.0910	0.0007
318.15	0.1557	0.2497	0.1980	0.1266	0.0942	0.1053	0.0018
328.15	0.1682	0.2887	0.2395	0.1422	0.1064	0.1212	0.0043
338.15	0.1812	0.3312	0.3037	0.1600	0.1181	0.1452	0.0098
Eutectic:water 50:50 (wt:wt)							
T (K)	Glycine	β-Alanine	L-Proline	trans-4-Hydroxy-L-proline	L-Lysine·HCl	L-Arginine	L-Cystine
298.15	0.0581	0.1371	0.2813	0.0515	0.1050	0.0214	0.0005
308.15	0.0743	0.1508	0.2952	0.0578	0.1205	0.0317	0.0013
318.15	0.0935	0.1714	0.3181	0.0707	0.1376	0.0501	0.0039
328.15	0.1112	0.1966	0.3401	0.0801	0.1574	0.0793	0.0073

Table S2. Sources and purities of amino acids used in this work.

Amino acid	Source	Purity (wt%)
Glycine	Sigma-Aldrich	>99.0
β-Alanine	Sigma-Aldrich	>99.0
L-Proline	Panreac	>99.0
trans-4-Hydroxy-L-proline	Sigma-Aldrich	>99.0
L-Lysine monohydrochloride	Sigma-Aldrich	>99.5
L-Arginine	Sigma-Aldrich	>98.0
L-Cystine	Sigma-Aldrich	>98.0

Table S3. Experimental data used for calibration curves of glycine.

Eutectic		***Eutectic:water 50:50 (wt:wt)***	
mass fraction of glycine (w)	refractive index (308.15 K, 0.1 MPa)	mass fraction of glycine (w)	density (g·cm^{-3}) (298.15 K, 0.1 MPa)
0.0000	1.44599	0.0000	1.150100
0.0202	1.44814	0.0249	1.157460
0.0399	1.44988	0.0508	1.165090
0.0601	1.45175	0.0751	1.172140
0.0798	1.45351	0.1000	1.179150
0.0996	1.45544	0.1249	1.186430

u(T)=0.01 K. u(P)=5 kPa. u(w)=0.0002. u(n_D)=0.0001 u(ρ)=0.0001

$$Refractive\ index = 0.0936 \cdot x + 1.4461 \quad (S.1)$$

$$R^2 = 0.9988$$

$$Density = 0.2903 \cdot x + 1.1503 \quad (S.2)$$

$$R^2 = 0.9998$$

Table S4. Experimental data used for calibration curves of β-alanine.

Eutectic		*Eutectic:water 50:50 (wt:wt)*	
mass fraction of β-alanine (w)	refractive index (308.15 K, 0.1 MPa)	mass fraction of β-alanine (w)	density ($g \cdot cm^{-3}$) (298.15 K, 0.1 MPa)
0.0000	1.44600	0.0000	1.150104
0.0302	1.44875	0.0500	1.160782
0.0600	1.45145	0.1001	1.170575
0.0916	1.45409	0.1500	1.180568
0.1202	1.45637	0.2002	1.190214
0.1498	1.45906	0.2500	1.199625

u(T)=0.01 K. u(P)=5 kPa. u(w)=0.0002. $u(n_D)$=0.0001 u(ρ)=0.0003

$$Refractive\ index = 0.0864 \cdot x + 1.4461 \quad (S.3)$$
$$R^2 = 0.9991$$

$$Density = 0.1976 \cdot x + 1.1506 \quad (S.4)$$
$$R^2 = 0.9996$$

Table S5. Experimental data used for calibration curves of L-proline.

Eutectic		*Eutectic:water 50:50 (wt:wt)*	
mass fraction of L-proline (w)	refractive index (308.15 K, 0.1 MPa)	mass fraction of L-proline (w)	density ($g \cdot cm^{-3}$) (298.15 K, 0.1 MPa)
0.0301	1.44841	0.0000	1.150104
0.0604	1.45110	0.1010	1.165964
0.0902	1.45360	0.1996	1.181118
0.1200	1.45632	0.3005	1.196699
0.1508	1.45891	0.4015	1.211770
0.1791	1.46152	0.4976	1.226005

u(T)=0.01 K. u(P)=5 kPa. u(w)=0.0002. $u(n_D)$=0.00006 u(ρ)=0.0003

$$Refractive\ index = 0.0877 \cdot x + 1.4458 \quad (S.5)$$
$$R^2 = 0.9962$$

$$Density = 0.1526 \cdot x + 1.1505 \quad (S.6)$$
$$R^2 = 0.9999$$

Table S6. Experimental data used for calibration curves of trans-4-Hydroxy-L-proline.

Eutectic		***Eutectic:water 50:50 (wt:wt)***	
mass fraction of trans-4-Hydroxy-L-proline (w)	refractive index (308.15 K, 0.1 MPa)	mass fraction of trans-4-Hydroxy-L-proline (w)	density (g·cm^{-3}) (298.15 K, 0.1 MPa)
0.0000	1.44579	0.0000	1.150251
0.0265	1.44854	0.0501	1.162772
0.0500	1.45077	0.0999	1.175436
0.0768	1.45342	0.1492	1.187954
0.1012	1.45571	0.1994	1.199934
0.1254	1.45800	0.2501	1.212897

u(T)=0.01 K. u(P)=5 kPa. u(w)=0.0002. u(n_D)=0.00007 u(ρ)=0.0002

$$Refractive\ index = 0.0971 \cdot x + 1.4459 \qquad (S.7)$$
$$R^2 = 0.9998$$

$$Density = 0.2502 \cdot x + 1.1503 \qquad (S.8)$$
$$R^2 = 0.9999$$

Table S7. Experimental data used for calibration curves of L-lysine·HCl.

Eutectic		***Eutectic:water 50:50 (wt:wt)***	
mass fraction of L-lysine (w)	refractive index (308.15 K, 0.1 MPa)	mass fraction of L-lysine (w)	density (g·cm^{-3}) (298.15 K, 0.1 MPa)
0.0000	1.44579	0.0000	1.150251
0.0203	1.44817	0.0512	1.158801
0.0402	1.45034	0.1000	1.166958
0.0600	1.45262	0.1495	1.175155
0.0801	1.45488	0.1999	1.183417
0.1002	1.45713	0.2495	1.191512

u(T)=0.01 K. u(P)=5 kPa. u(w)=0.0002. u(n_D)=0.0001 u(ρ)=0.0001

$$Refractive\ index = 0.1132 \cdot x + 1.4459 \qquad (S.9)$$
$$R^2 = 0.9999$$

$$Density = 0.1660 \cdot x + 1.1503 \qquad (S.10)$$
$$R^2 = 1.0000$$

Table S8. Experimental data used for calibration curves of L-arginine.

Eutectic		*Eutectic:water 50:50 (wt:wt)*	
mass fraction of L-arginine (w)	refractive index (308.15 K, 0.1 MPa)	mass fraction of L-arginine (w)	density ($g\cdot cm^{-3}$) (298.15 K, 0.1 MPa)
0.0000	1.44576	0.0000	1.150978
0.0203	1.44837	0.0252	1.154937
0.0400	1.45068	0.0505	1.159018
0.0600	1.45300	0.0757	1.162995
0.0801	1.45519	0.1003	1.167040
0.1001	1.45766	0.1251	1.170880

u(T)=0.01 K. u(P)=5 kPa. u(w)=0.0002. $u(n_D)$=0.0001 u(ρ)=0.00006

$$Refractive\ index = 0.1176 \cdot x + 1.4459 \quad (S.11)$$
$$R^2 = 0.9997$$

$$Density = 0.1599 \cdot x + 1.1509 \quad (S.12)$$
$$R^2 = 0.9999$$

Table S9. Experimental data used for calibration curves of L-cystine.

Eutectic		*Eutectic:water 50:50 (wt:wt)*	
mass fraction of L-cystine (w)	refractive index (338.15 K, 0.1 MPa)	mass fraction of L-cystine (w)	density ($g\cdot cm^{-3}$) (328.15 K, 0.1 MPa)
0.0000	1.43923	0.0000	1.132343
0.0039	1.43996	0.0040	1.133966
0.0064	1.44041	0.0080	1.135490
0.0086	1.44084	0.0120	1.137244
0.0121	1.44146	0.0160	1.138934

u(T)=0.01 K. u(P)=5 kPa. u(w)=0.0002. $u(n_D)$=0.00003 u(ρ)=0.00005

$$Refractive\ index = 0.1858 \cdot x + 1.4392 \quad (S.13)$$
$$R^2 = 0.9999$$

$$Density = 0.4111 \cdot x + 1.1323 \quad (S.14)$$
$$R^2 = 0.9997$$
